# Supplementary figures and images for: Long-read sequencing uncovers a complex transcriptome topology in varicella zoster virus
Source: BMC Genomics. 2018 Dec 4;19:873. doi: 10.1186/s12864-018-5267-8 (PMC6280550; doi:10.1186/s12864-018-5267-8)

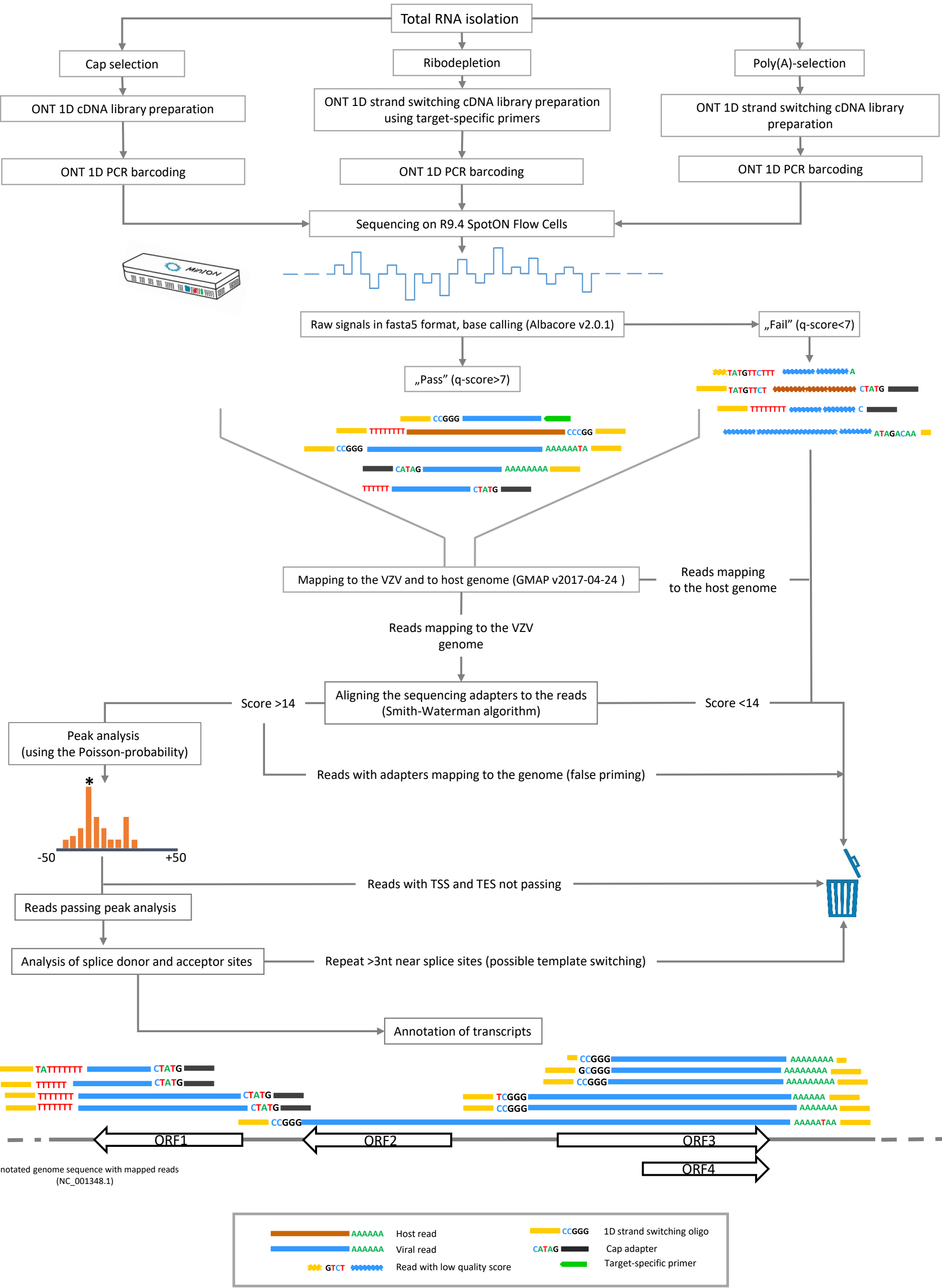

Supplement: Supplementary file 1 — The procedure of finding novel transcriptional isoforms. The flowchart describes the pipeline used to detect novel TSSs and TES-s. (PDF 505 kb) [file 12864_2018_5267_MOESM1_ESM.pdf]

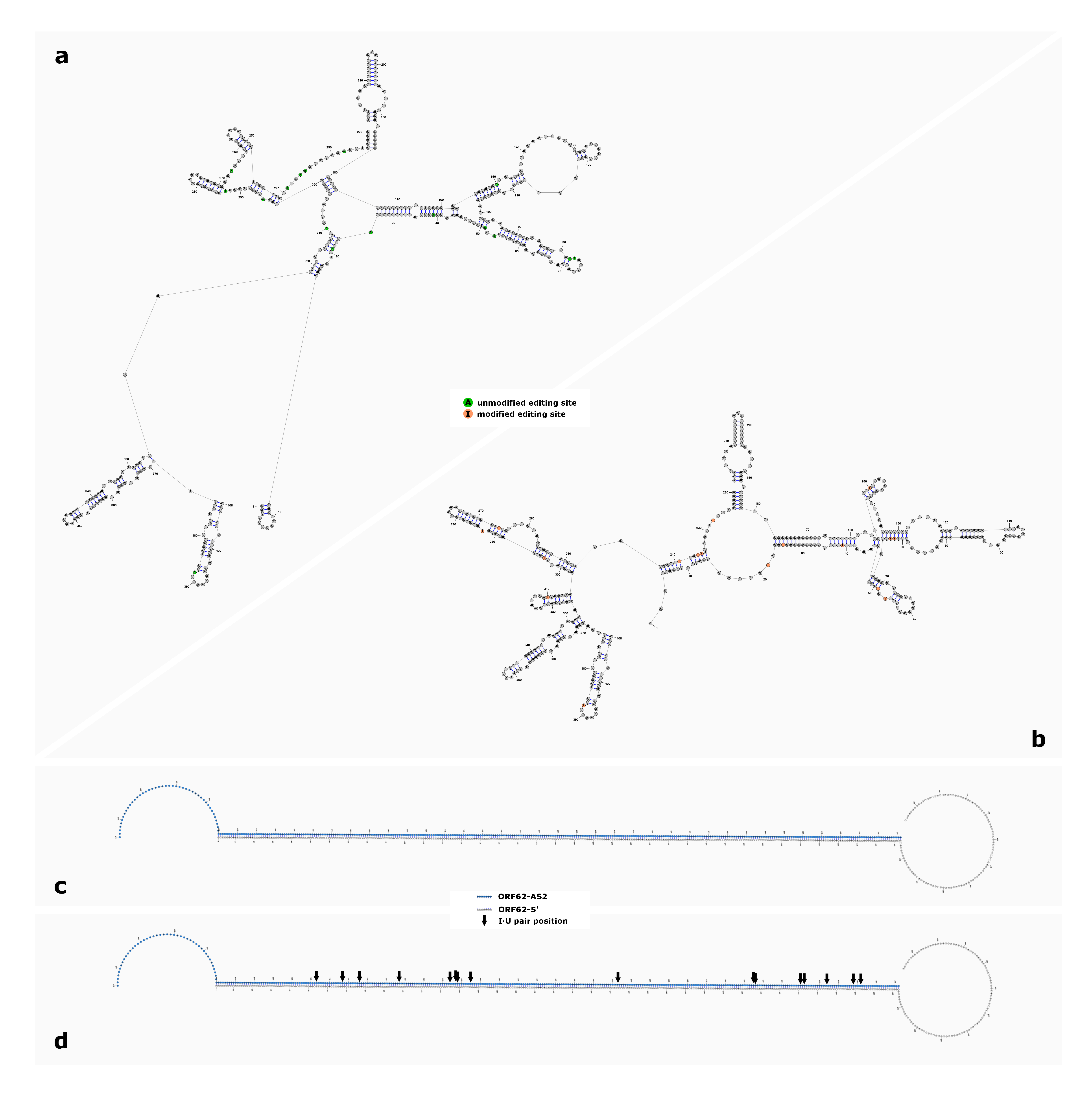

Supplement: Supplementary file 3 — The secondary structure of NTO3 (a. and b.) as well as the hybrid formed by the ORF62–5′ fragment and the NTO3 (c and d). a. The secondary structure of the unedited NTO3 with a free energy of − 143.2 kcal/mol. The adenines in the editing sites are colored in green. b. The secondary structure of the edited NTO3 with a free energy of − 169.4 kcal/mol. The inosines in the editing sites are colored in orange. c. The secondary structure of the sense-antisense hybrid composed of the first 467 bases of ORF62 labeled ORF62–5′ (gray) and the full sequence of NTO3 (blue), the latter is its unedited form. The free energy of the structure formed by the two molecules is − 822.9 kcal/mol. d. The secondary structure of the sense-antisense hybrid composed of the first 467 bases of ORF62 labeled ORF62–5′ (gray) and the full sequence of NTO3 (blue), the later in its edited form. The free energy of the structure formed by the two molecules is − 818.7 kcal/mol. The position of I·U base pairs is marked with black arrows. (TIFF 1530 kb) [file 12864_2018_5267_MOESM3_ESM.tiff]
